# Advances in Magnetic Nanoparticles Engineering for Biomedical Applications—A Review

**DOI:** 10.3390/bioengineering8100134

**Published:** 2021-09-30

**Authors:** Abdulkader Baki, Frank Wiekhorst, Regina Bleul

**Affiliations:** 1 Fraunhofer Institute for Microengineering and Microsystems IMM, Carl-Zeiss-Straße 18-20, 55129 Mainz, Germany; Abdulkader.Baki@imm-extern.fraunhofer.de; 2 Physikalisch-Technische Bundesanstalt, Abbestraße 2-12, 10587 Berlin, Germany; Frank.Wiekhorst@ptb.de

**Keywords:** magnetic nanoparticle synthesis, microfluidic systems, microreactor, magnetosomes, magnetic resonance imaging, magnetic particle imaging, magnetic fluid hyperthermia, drug delivery, magnetic actuation, micro/nanorobotics

## Abstract

Magnetic iron oxide nanoparticles (MNPs) have been developed and applied for a broad range of biomedical applications, such as diagnostic imaging, magnetic fluid hyperthermia, targeted drug delivery, gene therapy and tissue repair. As one key element, reproducible synthesis routes of MNPs are capable of controlling and adjusting structure, size, shape and magnetic properties are mandatory. In this review, we discuss advanced methods for engineering and utilizing MNPs, such as continuous synthesis approaches using microtechnologies and the biosynthesis of magnetosomes, biotechnological synthesized iron oxide nanoparticles from bacteria. We compare the technologies and resulting MNPs with conventional synthetic routes. Prominent biomedical applications of the MNPs such as diagnostic imaging, magnetic fluid hyperthermia, targeted drug delivery and magnetic actuation in micro/nanorobots will be presented.

## 1. Introduction

In 1959, Richard Feynman drew the attention of scientists to the significance of size and miniaturization of materials with his famous lecture *“There is plenty of room at the bottom”* [1,2]. After the starting gun had been fired, many methods were developed to manipulate atoms chemically to form nanoparticles and engineer nanomaterials. Subsequently, *the scientific community became fascinated with the enhanced functional properties of nanomaterials* compared to the corresponding bulk materials [3], and opening the door for plenty of technical and medical applications.

**Figure 1:**
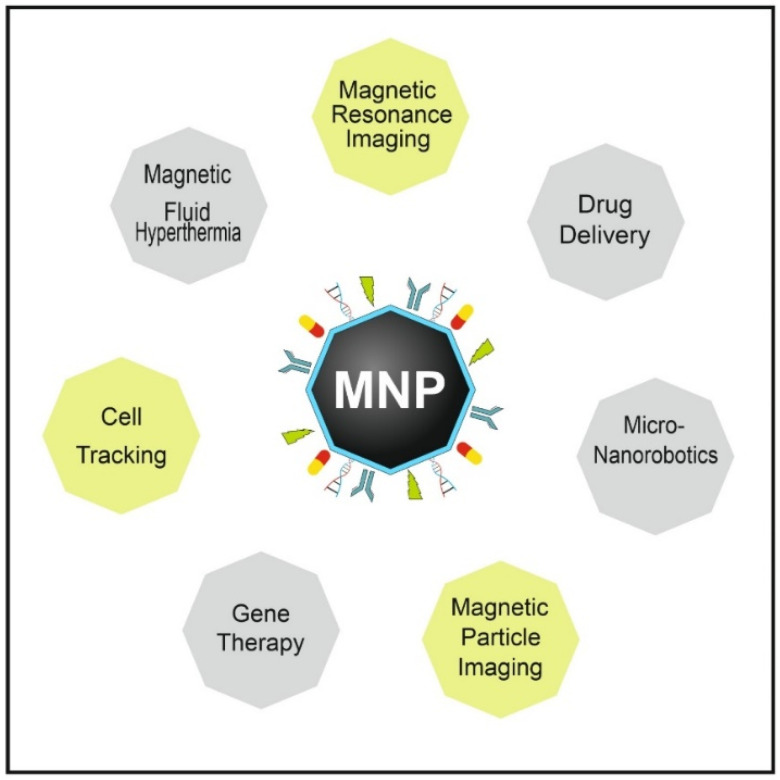
Biomedical application fields of MNPs. Due to the unique magnetic properties, the small particle diameter and the opportunity for additional functionalization with active substances connected to the surface, MNPs become ideally suited for diagnostic imaging (yellow: Magnetic Resonance Imaging, Magnetic Particle Imaging, cell tracking) and therapy (grey: gene transfection, drug-delivery, magnetic fluid hyperthermia, intervention by micro/nanorobots).

In this way, the unique properties of magnetic nanoparticles (MNPs) have been broadly studied for potential biomedical applications in the last decades. In particular, their magnetic properties strongly differ from bulk materials and become size-dependent [4,5]. A deeper understanding of the magnetic behavior of MNPs with respect to size was gained by applying domain theory [6], realizing that the behavior of magnetic material changes if the geometrical extension is reduced below a critical value, the so-called critical diameter *d*_cr_, which is normally a few tens of nanometers [7]. MNPs below this size only consist of one single magnetic domain, where all individual atomic magnetic moments of a MNP are uniformly coupled to exhibit a huge total magnetic moment. Above a certain temperature, thermal fluctuations permanently flip the magnetic moment of the MNPs into random directions so that no remnant magnetization will be measured for the MNP sample. Applying a magnetic field will (partially) align these moments leading to the strong magnetization of the MNPs that are exploited for the following applications: imaging, movement, heating or molecular sensing. MNPs with diameters > *d*_cr_ will comprise several magnetic domains, where inside each domain the individual magnetic moments are coupled and pointing in the same direction. By applying an external magnetic field, the structure of the domains can be altered, since it becomes energetically more favorable to form a larger domain with all moments aligned in the same direction. After removing the magnetic field, the MNP sample will exhibit remnant magnetization and show the typical hysteretic behavior, providing a powerful mechanism to produce heat in magnetic fluid hyperthermia. Additionally, MNPs with sizes between 10 nm and 100 nm were reported to be suitable for successful clinical application. While MNPs with diameters below 10 nm are removed by renal clearance from the body, MNPs above 100 nm are eliminated by macrophages, mostly after accumulation in the liver and spleen [8]. Besides size, MNP core morphology is crucial for medical applications. 1D-nanostructures like rods or tubes exhibit longer circulation times than spherical MNPs due to an opsonin-independent phagocytosis [8,9]. MNPs with high saturation magnetization enable lower doses, and therefore minimize undesirable side effects [10]. Thus, nanorods and nanocubes show enhanced performance in magnetic hyperthermia therapy over spherical counterparts due to higher magnetization saturation [10,11]. In addition, the hollow nanotubes can be exploited for drug loading inside and functionalization at the surface [12]. On the other hand, rod-shaped structures exhibit higher toxicity than sphere-shaped MNPs [13,14]. However, not only the size and morphology, but also size distribution and chemical composition of the MNP core and coating are relevant characteristics [15]. Hence, specific MNP types have been designed for and utilized in a broad range of applications (Figure 1) such as diagnostic imaging [16,17,18], targeted drug delivery [19,20], magnetic fluid hyperthermia [21,22] and combined applications thereof, called theranostics [23,24]. Every application requires tailored MNPs with specific magnetic and structural properties, for which reproducible and reliable synthesis approaches to manufacture high-quality MNPs are mandatory [25]. Additionally, synthesis parameters e.g., temperature, educts concentration, mixing ratios, solvents and surface ligands must be controlled and adjusted to produce suitable MNPs. For a successful translation into clinical applications, requirements on the scalability, reproducibility and biocompatibility of the process and resulting MNPs are further aspects of utmost importance [26,27].

**Figure 2:**
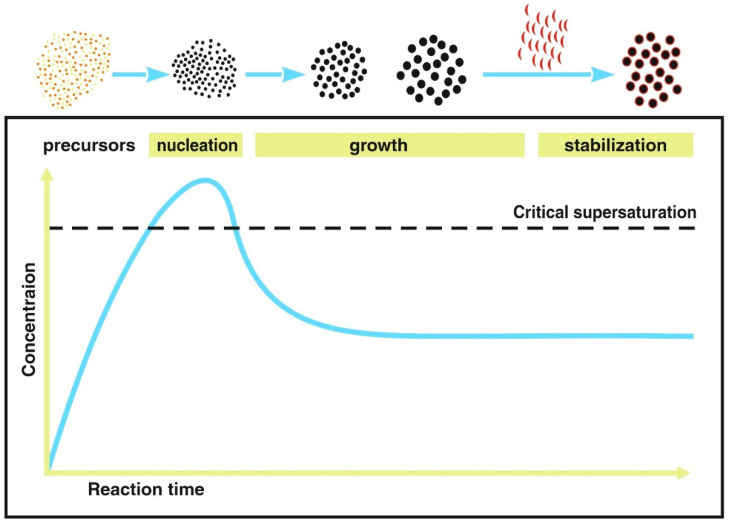
Steps during MNP synthesis in a bottom-up approach. When precursors reach supersaturation the nucleation of seeds occurs followed by growth. The stabilization of the MNP cores is carried out to prevent aggregation. Figure adapted from Ref [45].

In the last few decades, numerous bottom-up synthesis routes based on conventional batch processes have been developed, such as co-precipitation [28,29,30], sol-gel [31,32], ultrasonication [33], thermal decomposition [34,35], microemulsion [36,37] and microwave assisted synthesis [38,39], as well as top-down methods such as e.g., laser ablation and mechanical milling [40]. Because of the broad size and shape distribution of MNPs often produced by the top-down methods [41], bottom-up methods are preferable for medical applications. There, seed nucleation occurs when the precursors reach supersaturation (Figure 2). Subsequently, the particles grow by diffusion of solutes to the surface of the particles until a final size is reached, which is controlled by the solute concentration [42]. Stabilizing of the individual MNPs is crucial to prevent their aggregation to larger clusters [43]. During the nucleation and growth of the MNPs, factors such as surface energy, growth rate and temperature affect the final size, the size distribution, the crystal structure and the morphology, and thereby the magnetic properties of the product [43,44]. 

Co-precipitation according to Massart’s method [46] is the most commonly used and simplest conventional method for MNP production. Herein, a mixture of Fe^2+^/Fe^3+^ is precipitated by adding an alkaline, such as sodium hydroxide (NaOH) or ammonium hydroxide (NH_4_OH) at room or elevated temperatures (typically up to 100 °C). The pH-value of the reaction has a key role in controlling the properties of resulting MNPs [47]. Bhandari et al. presented a single step co-precipitation method for synthesizing curcumin functionalized MNPs that were employed to detect polychlorinated biphenyl 126, an inflammatory agent, in cell applications [48]. Thermal decomposition is another common MNP synthesis approach that relies on reactions of organometallic compounds, such as iron(iii) acetylacetonate Fe(acac)_3_, tris (acetylacetonato)iron(iii) (Fe(C_5_H_7_O_2_)_3_) or iron pentacarbonyl (Fe(CO)_5_) [6] at higher temperatures (typically 300 °C). Resulting MNPs consist of high-quality nanocrystals with narrow size distribution and uniformity in size and shape. Hyeon et al. fabricated cubic-shaped MNPs with sizes between 20–160 nm using Fe(acac)_3_, oleic acid and benzyl ether at 290 °C by varying reaction conditions [49]. A pioneer work on MNP production via thermal decomposition was presented by Krishnan group. By using iron(iii)-oleate as precursor and heating at 318 °C, they tailored polyethylene glycol (PEG)-coated MNPs with diameter of 26–28 nm and size and shape uniformity for cardio- and cerebrovascular imaging applications [35,50]. In most batch approaches for MNP synthesis, a sufficiently homogeneous mixing and uniform heat transfer cannot be achieved due to the large size of the reaction volumes, especially in scaled-up batches [25,51]. Therefore, the control and adjustment of seed and growth conditions are inevitably reduced. This disadvantage circumvents microfluidic synthesis, an approach that by minimizing the synthesis equipment to a small chip size, achieves higher and more rapid control of reaction parameters such as mixing ratio, temperature and heat transfer, resulting in increased MNP size and shape uniformity with a narrower size distribution compared to batch synthesis [25,43]. Another unique approach is employed by nature in the biosynthesis, using magnetotactic bacteria (MTB), with outstanding uniformity of size and shape [52,53,54].

**Figure 3:**
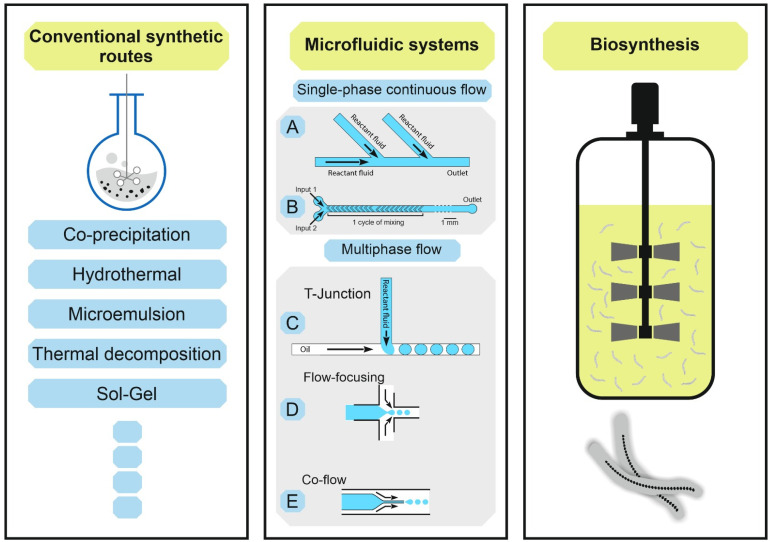
Synthetic routes of MNPs, left conventional synthetic routes in batch processes, middle microfluidic system with (A) homogenous continuous flow, (B) staggered herringbone mixer used in continuous flow (adopted from [100]), multiphase segmented flow (C) T-junction, (D) flow-focusing and (E) co-flow (adopted from [101]), right is biosynthesis using MTB in fermenter.

In the following, we review the latest developments in the synthesis of MNPs focusing on microfluidic methods. We compare those with conventional batch approaches and magnetosomes biosynthesis (Figure 3) regarding process requirements and efficiency for biomedical applications such as imaging, hyperthermia, drug delivery and magnetic actuation using micro/nanorobots.

## 2. Microfluidic Synthesis

In the last few decades, continuous flow processes, particularly using microfluidics have become a competitive and growing research field [55,56,57,58,59]. Scientists aim to optimize these methods to raise the quality of the produced MNPs and avoid typical drawbacks of conventional batch synthesis routes. Among others, these include inhomogeneous distribution of temperature, leading to hot spots that effect the reaction velocity locally and insufficient mixing, which cause concentration gradients. Both factors originate high batch-to-batch variability and a lack of reproducible product quality. As economic and ecologic drawbacks of conventional methods, e.g., the thermal decomposition method, high power demand due to reaction temperatures above 300 °C can be mentioned, as well as the use of organic solvents and toxic agents that might be present as undesirable residues in the final product [51,60,61,62,63]. Reaction routes in organic solvents are also generally time-consuming, as subsequent phase transfer to aqueous media is unavoidable before MNPs can act as imaging or therapeutic agents in biomedical applications. Microfluidic techniques have been discovered as promising approaches addressing the above-mentioned issues of conventional synthesis processes [64]. In microfluidic systems, the formation of products takes place in microchannels inside small devices called microreactors. The tiny paths increase the control of *reaction parameters* due to the high surface to volume ratio. *Resulting in the following advantages: sufficient mixing in millisecond range and improved (rapid) heat and mass transfer*. Additionally, the procedures offer other advantages such as flexible design and fabrication, fast change and screening of reaction parameters, cost efficiency, improved product quality, high throughput, higher reproducibility and the feasibility of automating the entire production process, including purification [27,65,66]. In contrast to conventional synthetic routes, continuous flow microreactors provide the separation of the two major steps during the formation of MNPs; (i) a rapid nucleation of the NP seeds occurs inside the microreactor, while the (ii) comparatively slow growth of NP takes place in the connected capillary, or ripening zone. Thus, a spatial and temporal separation of nucleation and growth can be achieved, leading to a high control of the particle formation process [67]. Generally, there are two main principles of mixing in the microreactor, (i) single-phase (continuous flow microfluidics) and (ii) multi-phase (droplet-phase or plaque flow microfluidics) [67,68]. In a single-phase or a continuous flow microfluidic system (Figure 3A), two or more miscible fluid streams containing the reagents flowing in a laminar stream are mixed in a homogenous phase by diffusion. Since the flow is laminar (turbulence free, e.g., Reynolds number < 10) [67], mixing is achieved by intermolecular diffusion. The mixing time is influenced by the flow rate and width of channels. A more effective mixing can be achieved at higher Reynolds numbers due to turbulent advection through the folding and stretching of fluid streams [67]. Technically, this can be implemented, e.g., by using staggered herringbone mixers (Figure 3B), enabling helical flows [69]. T-junction, Y-mixing, capillary, coaxial tubes and different designs of static micromixers are also utilized as microreactors in microfluidic particle formation processes. The phase-homogeneity offers reliable control of reaction parameters, such as temperature and reaction time, which makes continuous microfluidic synthesis suitable for both non-magnetic [67,70], as well as for magnetic nanoparticle production [71,72,73]. Furthermore, the technique is capable for multi-step syntheses and the subsequent modification of the product [74]. In another approach, the droplet-phase or segmented flow microfluidic synthesis, two immiscible phases, either gas-liquid or liquid-liquid (typically an oil phase and a water phase) form a droplet. The formed droplets containing the reactants work as tiny reactors and are transported in a segmented flow. In this way, variations in the residence time due to the parabolic flow in continuous flow profile can be reduced. However, the control of droplet formation and the homogeneity of droplet size are crucial. Moreover, droplet coalescence has to be avoided to provide the same reaction conditions in each droplet, and to ensure a reliable processing [75]. The generation of droplets in segmented flow can be achieved by several techniques, which include T-junction, flow focusing and co-flow [76,77]. As shown in Figure 3C, the droplet is formed in a T-junction by shear forces and liquid-liquid interfacial tension at the surface of the capillary. The liquid with the lower interfacial tension (than the capillary wall) will form a continuous phase, while the other liquid acts as a dispersed phase [75]. Capillary width and geometry, the flow rate and viscosity of the streams all influence the droplet formation [78]. The viscosity of the continuous phase, together with viscous drag forces versus the surface tension of the capillary, determine the break-up of droplets, and is therefore a significant parameter influencing the droplet formation [79]. In the second way of flow segmentation (see Figure 3D), flow focusing, the continuous phase is injected from two sides symmetrically, and combined with the dispersed phase of the central channel. After passage through a narrow orifice into the outlet capillary, stable droplets are formed [75,78]. Flow rate and geometric parameters of the setup influence the droplet characteristics [80]. In the third way, displayed in Figure 3E, a co-flow is used to produce segmented flow, where the dispersed phase is symmetrically enclosed by the continuous phase, both flowing in the same direction inside coaxial microchannels [81,82]. Segmented flow processing efficiently prevents the clogging and contamination of microchannels. Examples of MNP synthesis using segmented flow methods are reported in literature [83,84,85]. In contrast to continuous flow single phase processing, multistep reactions are challenging in segmented flow [67]. Moreover, to set up microfluidic processes for MNP synthesis successfully, numerous aspects have to be taken into consideration. These include: Flow profile inside the mixing structures as well as in capillary growth zones, capillary forces and material dependent surface effects, that can cause precipitation and agglomeration of MNPs on microwalls [86], leading to clogging of the capillary and finally process abortion [67]. Depending on the envisioned application, a careful material selection of the microfluidic device has to be performed. Photolithographic manufactured poly(dimethylsiloxane) (PDMS) microchips find broad application as the required equipment is easily available in many laboratories. However, their operation is limited regarding the process parameters such as flow rates, temperature and pressure. Usually, these chips can be run in a microliter per minute range. More resistant to pressure and temperature and suitable for higher flow rates are micromixers manufactured of stainless steel. However, the microstructuring of this inert material requires special and expensive microfabrication machining that is only available at specialized institutions and companies. Regarding possible throughputs and production scales, different approaches can be chosen. For *scale up through parallelization*, multiple micromixers are operated in parallel (or several parallel mixing structures are combined into one device), while for *internal scale up* the dimensions of the microchannels inside the microreactor are adjusted [87,88]. Scale-up through parallelization often lacks reliable processing, because as soon as one single channel is clogged, flow rates and flow profiles of all parallel mixers are disturbed. Consequently, the product quality immediately decreases, and the whole run has to be discarded. Hessel et al. enlarged the fluid inlets for an internal scale up and reached a flow rate of up to 8 L/h for liquids at the viscosity of water and a pressure of 1.5 bar [88]. Lin et al. reported the high mass production of 4.4 g/h of iron oxide MNPs in their microfluidic system [89]. The throughput of the method here depends on educt concentrations, flow rates, and the temperature of the synthesis, which determine the structure and magnetic characteristics of the product [25].

## 3. Magnetosomes Biosynthesis

An elegant biotechnological alternative to the chemical synthesis of MNPs is magnetosome biosynthesis using MTB, which was first discoveredby Bellini in 1963 and Blackmore in 1975 independently [90,91]. Magnetosomes are single-domain MNPs and membrane-enveloped [92,93]. The membrane is composed mainly of phospholipids and proteins [94]. In magnetosome biosynthesis, a variety of MTB are used as reactors for the formation of biomineralized crystals, which are aligned in chain-like agglomerates. In general, magnetosomes are uniform in shape and size within a specific strain but vary among different bacteria strains [52,53,54,95]. Magnetosome production depends on the cultivation of MTB for 36 to 60 h in complex media, supplemented with components that are essential for bacteria growth and magnetosome formulation such as yeast extract, minerals, ferric citrate, sodium lactate, magnesium sulfate and sodium thioglocate and ammonium chloride [96]. Additionally, a medium rich of iron ions, low dissolved oxygen concentration, neutral pH and moderate temperature range are some requirements for optimal biosynthesis [97]. The formation mechanism of magnetosomes is still not fully understood but can generally be divided into four major steps: (i) formation of the vesicle; (ii) magnetosome proteins are sorted to the vesicle membrane; (iii) iron is transported into the vesicle and mineralized as magnetite crystals; and (iv) magnetosomes are gathered in a chain-like structure and located for segregation during cell division. These steps of a complex process are controlled by over 40 genes, which encode the magnetosome-associated proteins. Thus, gen engineering and sequence modifications have key roles in synthesis optimization [53]. After cultivation, magnetosomes should be extracted from MTB to be used for medical applications. Four main extraction methods were reported to lyse bacterial cells including: (i) mixing MTB with 5 M NaOH; (ii) sonication; (iii) French press; and (iv) pressure homogenization [96]. After extraction, a careful purification of the magnetosomes is required to remove undesirable components such as surface proteins and potential immunogenic lipid components [98]. Magnetosomes bioproduction offers a powerful and sophisticated MNP system for biomedical applications. However, mass production (mass production in gram scale and cultivation time between 36 to 60 days [52]) remains challenging. Furthermore, extensive purification of magnetosomes from bacterial cell components are inevitable for in-vivo applications. The complexity of process design and development, as well as the relatively long preparation time for a new developed mutant, are some limitations that have to be addressed in further developments to increase industrial relevance. Studies aimed at a comprehensive understanding of the role of specific genes and their potential for process optimization are still ongoing [99].

## 4. Comparison of Different Syntheses

Recently, numerous techniques were developed to manufacture MNPs for different purposes (Figure 3). Conventional synthetic routes in batch are still dominant for many production processes. Although microfluidic and biosynthesis technologies promise enhanced production properties, especially for medical applications, they suffer from some drawbacks. In Table 1, we summarized the advantages and disadvantages of each technology.

## 5. Applications of MNPs

Magnetic nanoparticles have unique structural and magnetic properties that make them favorable as a tool for targeted transportation of active substances, generation of heat or local probe for imaging. In addition to their biocompatibility, stability, flexible surface modification, MNPs exhibit high magnetic moments that are utilized for biomedical applications [14,129,130]. Especially, iron oxide MNPs based on magnetite (Fe_3_O_4_) and maghemite (γ-Fe_2_O_3_) have been comprehensively studied. Resovist and Endorem are two examples of iron oxide MNPs that have been developed and applied as T_2_-weighted contrast agents for clinical magnetic resonance imaging [129,131]. Coating the surface of MNPs prevents aggregation in physiological tissue and bloodstream and enhances the biocompatibility. Often, it is a crucial step to prevent unwanted interactions of MNPs with their local biological environment as proteins and cells, and thus avoid their toxicity [132,133]. Commonly used coating materials are dextran [134,135,136] polyethylene glycol (PEG) [50,137] peptides [138] and serum albumin [132,139,140]. In this section, we present the latest developments in the translation of MNPs into biomedical applications like magnetic imaging, drug delivery, hyperthermia, and magnetic actuation.

### 5.1. Magnetic Imaging and Cell Tracking

Early diagnosis of diseases is advantageous in all treatment cases. Thus, imaging modalities have recently gained significant attention and are still developing. Magnetic resonance imaging (MRI) and magnetic particle imaging (MPI) are non-invasive imaging techniques that uses MNPs as contrast agents to deliver a high-resolution image without using ionizing radiation [132,141]. MRI detects the nuclear magnetic resonance signal of ^1^H atoms after applying radiofrequency pulses. Hence, tissue environment rich of water molecules will generate a different MR signal than a carbohydrate or fat rich environment, leading to contrasted images to discriminate between different tissues [142]. Magnetic contrast agents can shorten the *T_1_* (longitudinal) and *T_2_* (or transverse) relaxation time of surrounding water protons. Thus, signal intensity of *T_1_*-weighted images (positive contrast) will appear brighter and *T_2_*-weighted (negative) images will appear darker, leading to images with higher resolution. The relaxivities *r_1_* = 1/*T_1_* and *r_2_* = 1/*T_2_* are used to characterize the MNPs [18,143,144]. Ultrasmall iron oxide nanoparticles (USIO NP) were reported in various studies as *T_1_*-, *T_2_*- and dual-weighted contrast agents in in-vitro as well as in-vivo experiments [141,145,146,147,148,149,150,151]. Shen et al. manufactured exceedingly small magnetic iron oxide nanoparticles (ES-MIONs) with a core diameter *dc* = 3.6 nm by conventional co-precipitation and stabilization with polyacrylic acid (PAA). They resulted in *r_1_* = 8.8 and *r_2_* = 22.7 L·mmol^−^^1^s^−^^1^ and a ratio of *r_2_/r_1_* = 2.6 at a field strength of 1.5 T [152]. Whereas Besenhard et al. used continuous flow co-precipitation employing a millifluidic multistage flow reactor to produce dextran stabilized USIO NP. They obtained diameters of d_c_ = 5.4 (core diameter) and *d_h_* = 19 nm (hydrodynamic diameter) and higher relaxivity values *r_1_* = 10.7 L·mmol^−^^1^s^−^^1^ and *r_2_* = 36.9 L·mmol^−^^1^s^−^^1^ with a ratio of *r_2_/r_1_* = 3.4 at a field strength of 1.5 T [153].

In contrast, MPI directly detects the non-linear dynamic magnetic response of the MNPs exposed to an (sinusoidally) oscillating magnetic field. Additional magnetic field gradients are used for spatial encoding of the MNP distribution in the measured object. MPI provides high spatial resolution (below one millimeter) and excellent temporal resolution (1–10 ms) [154]. Theoretical calculations and experimental studies showed that optimized MNPs for MPI measurement are about d_c_ = 30 nm, which is not easily accessible by conventional synthesis routes [35,111]. The MPI performance of MNPs is characterized by the amplitude of the third harmonic normalized to the iron amount of the sample, A_3_* and the concentration-independent ratio between 5th and 3rd harmonic, *A_5_/A_3_*. Ferguson and Krishnan et al. reported 26–28 nm ±1.5 nm single-core MNPs with polyethylene glycol coating produced through thermal decomposition at 320 °C. The resulting MNPs have shown two to three-fold higher signal amplitudes compared to Resovist [35]. Resovist has been developed as MRI liver contrast agent and due to its good MPI performance became a gold standard for MPI measurements, even though it was withdrawn from the market, and they are not optimized for MPI [25].

Since MPI specifically detects the MNPs, the MP images are background-free, but do not provide any anatomical information. Thus, the combination of high-resolution 3D anatomical MRI data with molecular tracking of MNP tracers using MPI represents a promising hybrid MPI/MRI modality [155,156]. In a previous work, we presented our continuously synthesized MNPs via a micromixer in aqueous solution. After stabilization with tannic acid, the MNPs were coated with albumin which enhanced their colloidal stability in a physiological environment like a bloodstream. The MNPs exhibit diameters of *dc* = 27.7 nm and *d_h_* = 42 nm and relaxivity values *r_1_* = 6.2 L·mmol^−^^1^s^−^^1^ and *r_2_* = 600 L·mmol^−^^1^s^−^^1^, *r_2_* and a *r_1_/r_2_* ratio and for MPI, a higher value for A_3_* = 26 Am^2^/kg(Fe), *A_5_/A_3_* ratio compared to Resovist (Table 2), which makes these MNPs promising for clinical applications in the above-mentioned hybrid MP/MR imaging modality [132].

The magnetic performance of magnetosomes as potential contrast agents for MRI and MPI has also been studied [157]. Heinke et al. extracted magnetosomes and various mutants thereof from wild-type bacteria of the strain Magnetospirillum gryphiswaldense. They isolated magnetosomes with diameters of 36.5 nm for the wild type and of 23.0 nm to 44.2 nm for the mutants. Due to long range magnetic interactions in the larger crystallites, they formed chains and agglomerates. The *r_1_*- and *r_2_* relativities and ratio *r_2_/r_1_* of magnetosomes were determined at 0.94 T and 39 °C and showed higher *r_2_* relaxivities (*T_2_*-weighted) compared to Resovist (Table 2). The MPI measurements resulted in a 2.9- to 7.2-fold higher A_3_*-value compared to Resovist [158].

Additionally, MPI and MRI can be utilized for cell tracking. Wang et al. developed cubic-shaped MNPs (edge length = 22 nm, *d_h_* = 43 nm) for MPI to reveal in real time the migration and distribution pattern of transplanted bonemesenchymal stem cells given to hindlimb ischemia mice [159]. Song et al. coated 16 nm MNPs with a semiconducting polymer to fabricate the so called janus nanoparticles. After implanting these into mice, they showed efficient cell labeling and sensitive MPI tracking [160].

### 5.2. Hyperthermia

Hyperthermia is a powerful method for treating cancer cells by exposing tissue to elevated temperatures in a range of 42 °C to 48 °C. Since tumor cells are more sensitive to higher temperatures compared to healthy tissue, it can motivate either apoptosis (if the achieved temperature of the cells is below 45 °C) or necrosis (above 45 °C). Both apoptosis and necrosis have the capability to fight cancer cells with less damage of healthy human cells [161]. MNPs can be used to generate locally constrained heat at poorly accessible tissue regions by a magnetic fluid hyperthermia (MFH). An external alternating magnetic field of proper amplitude and frequency can be employed to generate heat by MNPs [162,163,164,165]. The enforced reorientation of the magnetic moments of the MNPs (either by the Néel mechanism of the moments inside the crystal structure or by Brownian rotation of the whole MNP) provides dissipative heat [166]. The efficiency of magnetic materials to generate heat in alternating magnetic fields is described by the specific absorption rate (SAR) or specific loss power (SLP). Besides frequency and amplitude of the applied magnetic field, the SAR strongly depends on structural and magnetic properties as shape, size distribution, crystallinity, saturation magnetization, anisotropy, relaxation time, concentration, and particle–particle interactions [167]. For larger MNPs (>100 nm), the main source of heat generated is hysteresis loss. Generally, large MNPs have higher saturation magnetization and therefore a larger hysteresis loop, leading to higher heating efficiency and heat generation [168]. As a disadvantage of the large size, an increased aggregation tendency might reduce the SAR and make the MNPs suboptimal for targeted delivery into tumor cells [169]. In smaller MNPs consisting of only one single magnetic domain, Néel and Brownian mechanisms are relevant for heat generation [163]. Different synthesis strategies were developed regarding size, shape and anisotropy with promising results [22,170,171,172]. Continuously synthesized MNPs showed remarkably high SAR-values and are promising candidates for hyperthermia treatment [111]. Another promising candidate for hyperthermia is magnetosomes. Their large core size and cubic shape structure results in large heat production of both individual magnetosomes, as well as chains made of them [173,174,175]. However, magnetic field amplitudes should be higher than 10 mT to fully exploit the advantage of magnetosomes, otherwise the losses of heat per cycle will be smaller than those of chemically produced MNPs [176,177]. Le Fèvre coated magnetosomes with poly-L-lysine after removing the endotoxins. Magnetosomes-poly-L-lysine lead to improved antitumor efficacy with complete tumor regression achieved in 50% compared to 20% for conventional MNPs in the treatment of glioblastoma in mice [178]. The work of Gandia et al. [179] showed that magnetosome chains are advantageous to enhance the hyperthermia efficiency than isolated magnetosomes, as investigated by Muela et al. [180].

For efficient clinical application, low doses of MNPs with high SAR value are favorable. Therefore, it is crucial to further understand and optimize the heat dissipation mechanism. Additionally, changes of the pH value, viscosity, and heat transfer of the surrounding environment of the living tissue should be taken into consideration [111].

### 5.3. Drug Delivery

By conjugation of MNPs with drugs, a powerful transport system becomes available that can even help to reduce undesirable properties of drugs like poor solubility, toxicity, nonspecific delivery and short circulation half-lives [129]. Thus, MNPs are attractive nanocarriers for targeted therapeutic drug delivery. Drug delivery can be achieved by two mechanisms. “Passive targeting” depends on the enhanced permeability and retention (EPR) effect. Generally, tumor growth is accompanied by the development of a surrounding leaky vessel system and therefore, MNPs can diffuse and accumulate within the tumor tissue [181,182]. “Active targeting” relies on guiding and accumulating the MNPs (and drugs) using externally applied magnetic field gradients [183], sometimes assisted by surface functionalization of MNPs with biomarkers [129].

Huang et al. produced MNPs via thermal decomposition and coated with Polyethylene glycol/Polyethyleneimine resulting in diameters *d_c_* = 9–14 nm and *d_h_* ≈ 67 nm. These MNPs were then conjugated with folic acid for diagnosis and treatment of breast cancer and loaded with Doxorubicin, an anticancer drug. The MNPs were tested to target a xenograft MCF-7 breast cancer tumor in nude mice. Due to a high relaxivity *r_2_* = 81.8 L·mmol^−1^·s^−1^), they could successfully be monitored by MRI [184]. Similar results were achieved by Yang et al. using heparin coated MNPs with diameters *d_c_* = 10 nm and *d_h_* = 125 nm that were conjugated with the chemotherapeutic agent Doxorubicin [185]. Huang et al. used a microfluidic chip to embed SPIO-NP (*d_c_* = 7 nm) into chitosan matrix and encapsulate Vinlastine. The composite resulted in well-defined spherical microparticles in a diameter range of 360 to 420 µm. The drug release of the chemotherapeutic agent was achieved by pulsatile external magnetic field [186]. Successful use of magnetosomes as nanocarriers was also reported by Long et al. Here, the chemotherapeutic agent Doxorubicin and asiRNA therapeutic agent were simultaneously conjugated to the magnetosomes using polyethyleneimine and succinimidyl 6-hydrazinonicotinate acetone hydrazone (SANH) as a bifunctional linker. Results showed that the Doxorubicin stayed stabile in normal pH blood environment and 40% of the drug was released at a pH-value of 5.5 after 280 h (pH-sensitive drug release). The nanocarrier was also capable to inhibit the proliferation of HeLa cells, and even to induce apoptosis [187].

A good prospect in clinical tumor treatment offers the combination of more than one method. Piehler et al. showed that functionalization of MNPs produced by conventional precipitation method with diameter of *d_c_* = 12 ± 3 nm with doxorubicin combined with magnetic fluid hyperthermia at 43 °C for 1 h results in tumor regression in vivo evidencing the increased therapeutic effect of the combination compared to the efficiency if either only magnetic fluid hyperthermia or intratumorally application of free doxorubicin has been carried out [188].

### 5.4. Gene Therapy

Gene therapy aims to treat diseases by transfer (or infection) of DNA or RNA sequences into targeted cells, generally by using viral vectors [189]. Alternatively, non-viral vectors can be used. Non-viral carrier systems offer crucial advantages for medical applications, such as stability, enzymatic degradation and low immunogenicity, as well as low toxicity and the ability to diffuse through cell membrane. Magnetofection is a non-viral method for transfection of nucleic acids using MNPs as carriers controlled by external magnetic fields [6,190]. For example, Zuvin et al. synthesized green fluorescent protein DNA-bearing polyethyleneimine-coated MNPs (average *d_c_* ≈ 30 nm und *d_h_* = 84 nm). On MCF7 human breast cancer cells, an increase of transfection efficacy after magnetic field exposure could be demonstrated [191,192]. Li et al. used magnetosome-like iron oxide nanochains to achieve gene transfection to mesenchymal stem cells to inhibit tumor growth of glioma mode rats [193]. Yang et al. fabricated galactose (Gal) and polyethyleneimine (PEI) MNPs (Gal-PEI-MNPs *d_h_* = 98.2 nm) to deliver siRNA to liver cancer cells and inhibited tumor growth in these cells [194].

### 5.5. Magnetic Actuation Using Micro/Nanorobots

An external magnetic field is a powerful means to remotely control the behavior of cells containing MNPs. Magneto-mechanical forces of the MNPs driven by an external magnetic field can destroy cells or cellular organelles in an anti-cancer treatment [195,196,197]. The mechanical forces of the MNPs are strong enough to destroy lysosomal membranes and lead to the permeabilization of membrane and subsequently initiate cell apoptosis [198,199,200]. Lunov et al. loaded clustered dextran coated MNPs with diameter *dc* ≈ 5 nm to liver cancer cells and achieved a lysosome-activated apoptosis by mechanical actuation using pulsed magnetic fields [201].

MNPs have been recently used to fabricate magnetic robots of micro- or even nanometer dimensions: These small-scale devices are intended to minimize invasive procedures in surgery or to avoid exposure to radiation [202]. Magnetic micro/nanorobots consist of two components, a biotemplate, a flexible part often in a shape of helix or filament to enhance the mobility in physiological fluids like bloodstream and a magnetic component containing MNPs for magnetically driven actuation by magnetic field gradients [203].

Magnetotatic bacteria are a natural example of nanorobots that can be used for drug delivery. Felfoul et al. transported in-vivo drug-loaded nanoliposomes into hypoxic regions of a tumor using magnetococcus marinus bacteria (strain MC-1) [204]. Another example is biohybrid magnetic robots as reported by Yan et al. fabricated from spirulina microalgae as a biological matrix via a facile dip-coating of MNPs. The movements of a swarm of the microrobots (microswimmers) inside rodent stomach have been successfully tracked using MRI [205]. Alapan et al. reported bacteria-driven microswimmer using red blood cells as autologous carriers for guided drug delivery. Red blood cells loaded with doxorubicin and MNPs were fixed on the Escherichia coli MG1655 via a biotin-avidin-biotin binding complex, and the microswimmers were directed using an external magnetic field gradient. After the treatment, the bacteria were removed using the on-demand light-activated hyperthermia [206].

### 5.6. MNPs in Theranostic Applications

In the last decades, theranostic nanomaterials have emerged that combine therapeutic components with diagnostic imaging capabilities of MNPs. They are promising for theranostic applications due to their biocompatibility, biodegradability, and surface modification capabilities. For diagnosis, the MNPs are tracers in imaging and cell tracking, while for therapeutic applications, their hyperthermia and drug delivery properties are utilized. Cho et al. demonstrated the assembly of 20 nm cubic MNPs (produced by thermal decomposition) into larger nanostructures up to 100 nm using serum albumin. The assembly showed high *r_2_* relaxivity (~500 L·mmol^−^^1^·s^−^^1^ at 1.41 T) in MRI and were successfully detected after injection into mice bearing U87-MG tumor cells. Additionally, tumor growth reduction was achieved by magnetic hyperthermia treatment [207]. A combination of MPI and drug delivery in vivo was presented by Zhu et al. They prepared nanocomposites of poly(lactide-co-glycolide acid) and MNPs (PLGA-MNPs) nanoclusters loaded with doxorubicin. The nanoclusters induced gradual decomposition in tumor environment at pH = 6.5. The disassembly of the iron oxide core cluster (detected by MPI) and the release rate of the drug over time showed linear correlation (R^2^ = 0.99) [208]. Lu et al. developed MRI-visible nanocarriers using MNPs to monitor the targeted delivery of siRNA to neuronal stem cells, and at the same time, to direct their neuronal differentiation through gene silencing in stroke therapy. Additionally, an improvement in recovery of neural function from ischemic strokes in rats was achieved [209].

## 6. Clinical Translation of MNPs

In 2009 already, Ferumoxytol (Feraheme), a MNP-based drug capped by polyglucose sorbitol carboxymethyl ether [210], was approved by the US Food and Drug Administration (FDA) for treatment of iron deficiency anemia in adult patients with chronic kidney disease (CKD) [211]. Moreover, since Ferumoxytol is uptaken by macrophages, it can be applied for imaging of macrophages, tumors or vascular lesions by MRI [212]. Magforce AG developed aminosilane-coated MNPs to treat solide tumors locally by hyperthermia. The MNPs can be presented to tumor directly or into the resection cavity wall. Subsequently, tumor cells are destroyed or become more sensitive to radiotherapy or chemotherapy. Currently, two centers in Germany started to commercially treat brain tumor patients and further clinical studies are under review by the FDA [213]. However, although several studies have demonstrated successful preclinical applications, many factors hinder the implementation of MNPs in versatile theranostic applications. These include high process complexity, high cost and long tumor treatment trial period, low drug delivery accumulation of MNPs in the target region and the possible lack of enhanced permeability and retention (EPR-effect) in a human solid tumor compared to mouse models [214]. However, the most significant factors preventing clinical translation are toxicity and safety of MNPs. MNP toxicity can be associated with toxicity of the precursor(s) used for preparation, coating, chemical composition, oxidation state of MNPs, protein interaction and high dosage [215,216]. Therefore, further improvements in these fields are required for the safe clinical translation of MNPs.

## 7. Conclusions

Magnetic nanoparticles have become an attractive and increasingly important part of diagnostics and therapeutic treatment of diseases. They are widely investigated and developed for a broad range of biomedical applications, each using one or more of their magnetic properties to generate a specific effect that is controlled from outside by magnetic fields. The wide variety of applications demonstrate the significance, but at the same time the need for reliable, reproducible and on top economic as well as ecological methods for successful translation into clinical applications.

Nevertheless, many challenges remain in finding and engineering an ideal magnetic nanoparticle system for an envisaged biomedical application. This is reflected in the major efforts still ongoing in further developing synthesis methods of magnetic materials. Although considerable achievements have been made in these synthesis approaches, there still is huge demand for advanced synthesis methods. With microfluidic synthesis and biosynthesis of magnetosomes, two advanced techniques have been presented, both very powerful approaches to provide magnetic entities with outstanding structural and magnetic quality.

The actual state of extensive research on microfluidic synthesis methods of MNPs and the advantages over conventional (batch) synthesis methods have been discussed above. However, looking at the MNPs presently in biomedical applications as presented in Section 5, it is striking that mostly all diagnostic and therapeutic approaches rely on MNPs that have been synthesized by conventional synthesis methods. The reason for this is assumed to be constraints in the microfluidic approach regarding clogging of the reactor, sufficient throughput, effective purification strategies, GMP-compliant production, or scalability.

Aqueous synthesis as a method to continuously produce single core MNPs without immunogenic membrane and endotoxins is a very attractive approach, especially if combined with in line purification and in line process control. Thus, this straightforward, fast, and efficient approach additionally offers a high automation potential. However, in order to reach the MNP quality as provided in biosynthesis of magnetosomes, further optimization is required.

Although MNPs hold great promise in biomedical applications, there are still problems that have to be solved before the translation into clinical settings becomes feasible. One of the major challenges are the biocompatibility and the toxicity of the MNPs in the long term. Further detailed and comprehensive studies are required to resolve the effects of composition, morphology, size, shape, and structure of MNPs on their clearance and fate from a living organism. Further advancing techniques such as continuous microfluidic synthesis and biosynthesis will make a significant contribution to tailor MNPs for safe and effective clinical applications.

## Figures and Tables

**Table 1:** Comparison of conventional, microfluidic systems and biosynthesis of MNPs.

Parameter	Conventional Batch Methods	Microfluidic Systems	Magnetosome Biosynthesis
surface to volume ratio	about 100 m^2^/m^3^ [51,102]	10,000–50,000 m^2^/m^3^ [51,102]	-
mixing efficacy	mechanical stirring takes minutes to reach homogeneity [63]	homogenous, tunable, efficient, <60 ms [70,103,104,105,106]	-
heat transfer	heating plate, heterogeneous, often require high temperature [25]	microchannels enable homogenous and rapid heat and cool transfer, small heat amount [67,70,86,103,105]	-
energy resource	conventional	conventional	ATP-based [52]
residence time	several hours to days	controllable and tuneable from seconds to minutes [25]	cultivation within 36 and 60 h [96]
separation between nucleation and growth stages	poor due to inhomogeneous mixing and heat transfer [25,51]	nucleation in the microreactor and growth in dwell zone [25,67,107,108,109]	nucleation in vesicle and the iron ions are transferred from the surrounding environment, protein-associated [53,54,110]
reaction time	minutes—hours [43]	seconds [25,86,105,111]	Several days to weeks [25,93,112]
control of reactions parameters	poor, except for thermal decomposition [50]	high due to efficient heat and mass transfer [67,103,105]	suitable environment required for bacteria growth [52,98]
reagent volume	millilitre to litre [44]	micro to nanolitre [44]	litre
purification	mandatory if solvents are used for phase-transfer and biocompatible coating [25]	on-line integration possible, e.g., Tangential Flow Filtration (TFF) [113]	magnetic separation, ultrasonication and removal of proteins, nucleic acids and lipopolysaccharides are mandatory to reduce immunotoxicity [98,114]. Coating (for example by poly-l-lysine) to obtain stable nonpyrogenic MNP suspension [115]
product homogeneity	quality reduction by concentration gradients and hot spots in the reaction flask [25,51]	enhanced quality due to homogeneous morphology, narrow size distribution [25,67,116]	high within one bacteria strain but strain variation possible [52,53,54,95]
reproducibility, production rate and scale-up capability	significant batch to batch variations in size, morphology, and magnetic properties [25,111,117,118,119], poor scaling up capability. A reported study from Lin et al. showed a production rate of 4.73 g/h for microfluidic synthesis comparing to 1.4 g/h for conventional synthesis with the same conditions [89]	continuous production, no batch-to-batch variation, high scale-up capability	high at the defined environmental conditions [92], mg/(L · day) production rate [52], high scale-up capability, though challenging due to long term bacteriostatic growth conditions [38,40,46,78]
clogging	not applicable	microchannel-wall blocking during nucleation or by agglomeration [77,104,120,121,122]	not applicable
automation	poor	feasible/integratable [66,123,124]	-
capability of on-line characterization	not applicable for batch, though magnetic characterization of whole batches by magnetic particle spectroscopy is feasible	parameter control and synthesis adjustment feasible during synthesis, control of magnetic parameters by magnetic particle spectroscopy [25,125] and NMR [126]	-
cost	low, common lab equipment	expensive microreactor fabrication	expensive specialized equipment [112]
raw material and energy consumption	high, some reactions require organic solvents for phase-transfer to aqueous phase Some reactions are performed at temperatures above 320 °C [50,70,86,127]	aqueous synthesis at moderate temperatures feasible, raw materials and energy consumption can be saved [70,86,127]	sterile raw materials and cell cultivation materials required, temperature control during the bioproduction for days [52,53,54]
usability for medical applications	possible, long fabrication times, post-treatment and phase-transfer from organic solvents may be required [25]	possible, capable for sterile production, no FDA approved process yet [25]	possible due to biosynthesis, purification required to remove lipopolysaccharides [52,128]

**Table 2:** Comparison of MNP properties for magnetic imaging (MRI, MPI) produced via microfluidic synthesis, conventional batch synthesis and biosynthesis. System information, coating and measurements field strength are given in the three columns to the left, followed by magnetic properties. Note, the specific non-linear dynamic susceptibility A_3_* was determined by MPS at f_e_ = 25 kHz and an amplitude of B_e_ = 25 mT. All relaxivities are stated for a field strength of 1.5 T, except for the last three systems, wild type, mutant-3 magnetosomes and Resovist as standard-measurement to magnetosomes, which were measured at 0.94 T.

Sample System	Synthesis Approach	Coating	*Dc* *nm*	A_3_*Am^2^/kg (Fe)	*r*_1_ L/(mmol·s)	*r*_2_ L/(mmol·s)	*r*_2_/*r*_1_	Ref
ES-MIONs	conventional co-precipitation	polyacrylic acid (PAA)	3.6	-	8.8	22.7	2.6	[152]
USIO NP	microfluidic multistage flow reactor	dextran	5.4		10.7	36.9	3.4	[153]
Single core MNPs	conventional thermal decomposition	Polyethylene glycol	26–28	26	-	-	-	[35]
Single core BSA-coated	continuously synthesized via micromixer	bovine serumalbumin	27.7	26	6.2 (4)	600 (10)	97	[132]
Resovist, multi-core, bimodal size distribution mean cluster size 24 nm	conventional	carboxydextran T1.8 kDa	6	8.7	7.4 8.7	9561	15	[132]
Wild type	MTB	Lipid bilayer	36.5	25–63	10.3	457	44.4	[158]
Mutant-3	MTB	Lipid bilayer	32		12.5	594	47.5	[158]
Resovist	conventional	carboxydextran	6		20.0	219	11.0	[158]
